# The impact of COVID‐19 on the resident well‐being in a single US healthcare system

**DOI:** 10.1002/hsr2.392

**Published:** 2021-10-01

**Authors:** Haseeb Chaudhary, Zohaib Yousaf, Anthony Donato

**Affiliations:** ^1^ Department of Medicine Tower Health West Reading Pennsylvania USA; ^2^ Department of Medicine Hamad Medical Corporation Doha Qatar; ^3^ Clinical Research Dresden International University (DIU) Dresden Germany

## ETHICS STATEMENT

This work is original, has not been, and is not under consideration for publication in any other journal. All authors have reviewed and approved the final version of the manuscript. The study was approved by the Institutional Review Board of Reading Hospital, Tower Health Healthcare system, Pennsylvania, PA.

## 1 INTRODUCTION

The coronavirus disease 2019 (COVID‐19) pandemic has impacted the entire society, but arguably, the healthcare system the most.^1^ Challenges to providers include rapidly evolving treatment paradigms, the risk of personal infection or spread to loved ones, shortages of personal protective equipment (PPE), extreme workloads, and added childcare responsibilities with school closures..^1^

Resident physicians form the bulk of the frontline physician healthcare workforce. They are the most junior and most vulnerable group of physicians.^2^ Therefore, policies and procedures exist to provide a safe and abuse‐free culture for residents.^3^ In an article titled “Legacy of Abuse in a Sacred Profession: Another Call for Change,” Janet Rose Osuch discusses the high workload and the prevalent verbal and psychological abuse of residents globally.^4^ This abuse is also described in several opinion pieces like the “COVID‐19 Crisis Exposes Resident Abuse.”^5^ A pandemic can have stressors compounded.^6^ Direct exposure to sick patients has also been associated with higher reported stress and burnout levels.^7^

A physician's well‐being directly impacts their competence, professionalism, career satisfaction, and the quality of care delivered to their patients.^8^, ^9^ We sought to understand better the pandemic's impact on the well‐being of residents. We hypothesized that well‐being would likely worsen during the pandemic and may be affected by PPE availability, training on its use, and direct exposure to COVID‐19 patients.

## 2 RESEARCH DESIGN AND METHODS

### 2.1 Study design

A cross‐sectional survey of US residents was performed between July and August 2020 at a six‐hospital south‐eastern Pennsylvania health system.

### 2.2 Inclusion criteria

Participants surveyed included 257 residents from 20 different residencies across the system's six hospitals. Convenience sampling was used, and the survey was conducted using email. A statement describing the purpose of the survey was included in the email. All results were de‐identified before analysis.

### 2.3 Exclusion criteria

Residency programs with less than five respondents were excluded.

### 2.4 Study parameters

The Mayo Well‐Being Index (WBI) is a 7‐point scoring tool that correlates well with the mental quality of life (QOL), fatigue, and suicidal ideation.^10^ The seven questions are dichotomously scored as “yes” or “no,” describing a specific facet of low well‐being, with higher scores indicating poor well‐being. A score of ≥5 is considered a high score indicating the low mental QOL, high fatigue, or recent suicidal ideation.^11^ WBI is validated for the residents working in the United States of America.^10^ Additional questions included demographics, training level, exposure to severe acute respiratory syndrome coronavirus 2 (SARS‐CoV‐2)‐positive patients, training for doffing/donning of PPE, adequate PPE availability, and involvement in care for patients with COVID‐19 patients. After completion of the survey, participants were offered self‐help resources, and the resources accessed were analyzed.

### 2.5 Statistical analyses

A descriptive and summary analysis was performed. The analysis compared mean WBI between residents surveyed 1 year before the COVID‐19 pandemic and residents working during the first wave of COVID‐19. The means were compared with the national average. Answers to PPE, training on that equipment, exposure to patients with COVID‐19, and accessed resources were reported as percentages.

## 3 RESULTS

Surveys were sent to 257 participants, out of which 140 recipients (54.4%) signed up to participate in the WBI survey. The total number of completed responses was 35% (90/257), of whom 55% (45/90) were female. The mean 7‐point WBI score for all participants was 2.42 (standard deviation [SD] 1.83), an 18.2% raw difference, with lower scores indicating better well‐being than scores performed in the same system during the same months in 2019. Male residents had a higher mean WBI score of 2.59 (SD 1.96) than females (Table 1).

**TABLE 1:** Mean well‐being index (WBI) by gender

Sex (n)	WBI (SD)	National WBI comparison 2020 (SD)	% difference vs national	Sex (n)	WBI (SD)	% change, 2020 vs 2019
Female (49)	2.23 (1.75)	2.82 (1.94)	−20.92%	Female (26)	3.3 (1.64)	−32.42%
Male (38)	2.59 (1.96)	2.25 (2.00)	+15.11%	Male (21)	2.19 (1.76)	+18.26%

Abbreviation: SD, standard deviation.

**FIGURE 1:**
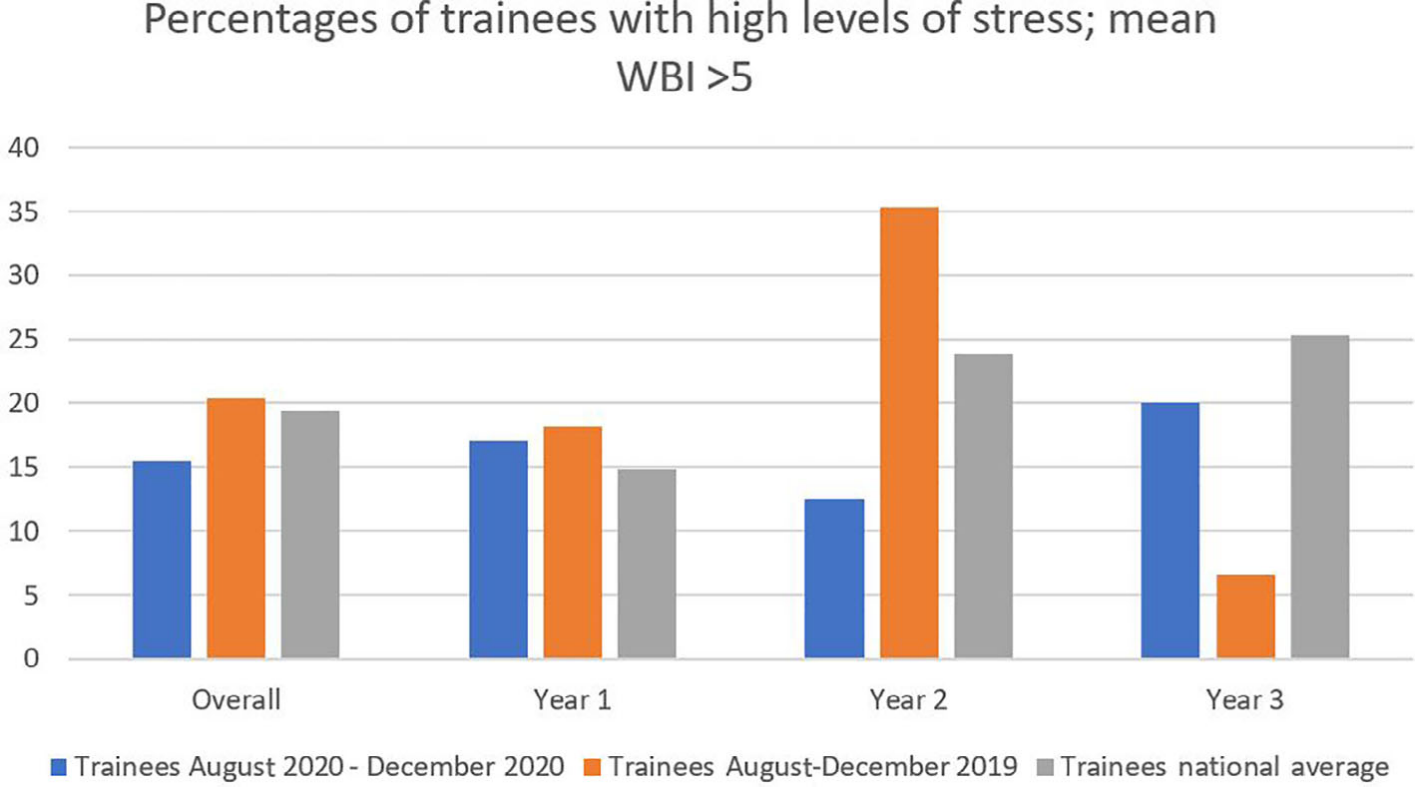
Percentage of participants with high levels of stress mean score > 5.0

**FIGURE 2:**
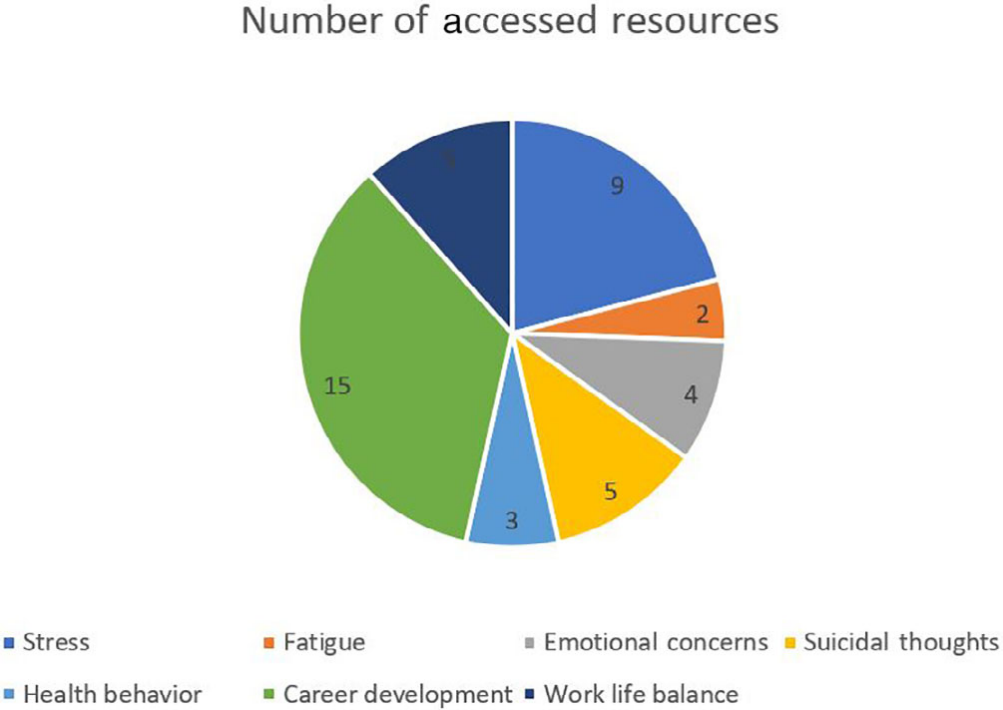
Number of accessed resources

Third‐year residents in our survey had the highest mean score of 2.95 (SD 1.77). The percentage of residents with a mean well‐being index score > 5 was 15.8% (Figure 1). However, 20% of postgraduate year‐3 residents (n = 18) had scores >5 (20%). Ninety‐two percent of surveyed participants reported adequate training in donning and doffing PPE, and 81% reported PPE availability. The most commonly accessed resources following the survey completion were on career development (n = 13) and stress and anxiety (n = 8) (Figure 2) (Table S1).

## 4 DISCUSSION

Contrary to our hypothesis, our survey results showed improved overall trainees' mean WBI scores compared to the prepandemic survey and lower rates than the national US average. Male residents had a higher score as compared to the national comparators and last year's scores. Female respondents' average WBI was lower than national comparators. Last year's third‐year residents had an 8% higher score than the prepandemic survey and 3% higher than the national average (Table 2).

**TABLE 2:** Comparison of mean well‐being index (WBI) scores by year of training

2020 Residents (n = 90)			2019 Residents (n = 50)	
Year in Residency	Mean WBI (SD)	National mean WBI (SD)	Year in Residency	Mean WBI (SD)
Year 1 (44)	2.61 (1.94)	2.28 (1.88)	Year 1 (21)	2.82 (2.01)
Year 2 (23)	2.13 (1.67)	2.87 (2.04)	Year 2 (16)	3.35 (1.75)
Year 3 (18)	2.95 (1.77)	2.87(2.05)	Year 3 (13)	2.73 (1.57)
Overall	2.44 (1.87)	2.53 (1.99)	2.96 (1.84)	

Abbreviation: SD, standard deviation.

The majority (81%) of the participants had adequate PPE availability, and 92% of respondents had appropriate training in donning and doffing techniques. Our male participants showed higher scores (worsened well‐being) compared to the national average. The finding contrasts with the national data, in which higher scores (worse well‐being) were reported in female residents.^11^

Our study also found that 16% of the participants had a mean well‐being index score above 5, which correlates to high stress levels. However, this was comparatively less than the previous local survey, mean national average in the 2020 (19.4%), staff physicians (35.1%), nurses (57.1%), students (22.0%), and other healthcare employees (46.3%).^11^ Stress levels in PGY‐3 residents were 8% higher than the prepandemic survey and 3% higher than the national average (Table 2). Career development was the most accessed module (Figure 2). We speculate that a higher level of critical decision‐making, searching for work, and fellowship placement may have contributed to this subset's higher scores.

Most of our participants (73%) had direct exposure to COVID‐19 patients. As the survey was anonymous, we could not extract data based on COVID‐19 responses and tabulate them against the mean WBI scores.

Strengths of this work include using the same well‐being index as a validated tool for self‐assessment screening purposes. The previous surveys provided a fair comparison to the residents' pre‐COVID‐19 mental well‐being in our health system. However, our study has some limitations. Our sample was female predominant (55%). In a typical 3‐year residency, 33% of the residents graduate each year, so our comparison populations differ. This study was carried out just after the first peak of the COVID‐19, so the real impact of the pandemic on well‐being may be unmasked after a prolonged period of pandemics with repeated weaves of COVID‐19. Interpretation of subgroups in our study (men, PGY‐3s) must be made with caution, as a few results can skew means in small sample sizes. Although our response rates (35%) were higher than the nationally reported rates for the WBI (22.5%), there could be very significant low well‐being rates among those who did not participate that were missed by our sampling.

## 5 CONCLUSION

Our survey showed lower than expected levels of poor well‐being in the resident physicians during the pandemic's first wave. The higher stress levels were noted in the final years of residency, a finding that may warrant additional institutional efforts to counteract the burnout and fatigue among the graduating residents. When facing novel healthcare threats, adequate equipment availability, adequate training, and a well‐managed patient care burden can positively impact physicians' most vulnerable group, that is, resident physicians' well‐being.

## CONFLICT OF INTEREST

None of the authors has any conflict of interest to disclose.

## AUTHOR CONTRIBUTION

Conceptualization: Haseeb Chaudhary, Zohaib Yousaf.

Investigation: Haseeb Chaudhary.

Methodology: Haseeb Chaudhary, Zohaib Yousaf.

Project administration: Haseeb Chaudhary, Zohaib Yousaf.

Supervision: Anthony Donato.

Writing—Original Draft Preparation: Haseeb Chaudhary.

Writing—Review and Editing: Zohaib Yousaf, Anthony Donato.

All authors have read and approved the final version of the manuscript.

Haseeb Chaudhary (lead author) had full access to all of the data in this study and takes complete responsibility for the integrity of the data and the accuracy of the data analysis. The lead author Haseeb Chaudhary affirms that this manuscript is an honest, accurate, and transparent account of the study being reported, that no important aspects of the study have been omitted, and that any discrepancies from the study as planned have been explained.

## CONSENT

Informed consent was not required, as this is an anonymous survey conducted after approval from the institutional review board.

## ROLE OF STUDY SPONSOR OR FUNDER

The study sponsor/funder was not involved in the study's design, the collection, analysis, and interpretation of data writing the report and did not impose any restrictions regarding the report's publication.

## Supporting information

**Table S1.** Additional survey questions specific to coronavirus disease 2019 (COVID‐19).

## Data Availability

The data that support the findings of this study are available from the corresponding author upon reasonable request.
