# Supplementary material for: The impact of COVID‐19 on the resident well‐being in a single US healthcare system
Source: Health Sci Rep. 2021 Oct 1;4(4):e392. doi: 10.1002/hsr2.392 (PMC8485623; doi:10.1002/hsr2.392)
Supplement: Supplementary file 1 — Table S1. Additional survey questions specific to coronavirus disease 2019 (COVID‐19). [file HSR2-4-e392-s001.docx]

**Table s1: Additional survey questions specific to COVID-19**

| **Question:** In what capacity have you been interacting with suspected or confirmed COVID-19 patients? |
| --- |
| \| **Option** \| **Count** \| **Percent** \| \| --- \| --- \| --- \| \| No interaction \| 7 \| 7.8% \| \| Only virtually via telephone or video \| 17 \| 18.9% \| \| Full interaction with virtual and face to face encounters \| 66 \| 73.3% \| |
| Do you have adequate personal protective equipment (mask/gown/gloves) available? |
| \| **Option** \| **Count** \| **Percent** \| \| --- \| --- \| --- \| \| Yes \| 72 \| 80.9% \| \| No \| 17 \| 19.1% \| |
| **Question:** Do you have adequate training regarding donning/doffing (wearing) PPE? |
| \| **Option** \| **Count** \| **Percent** \| \| --- \| --- \| --- \| \| Yes \| 83 \| 92.2% \| \| No \| 7 \| 7.8% \| |
